# AI-driven voltage map analysis for optimizing catheter ablation strategy in atrial fibrillation: a proof-of-concept study

**DOI:** 10.1093/ehjdh/ztag054

**Published:** 2026-03-31

**Authors:** Takeshi Tohyama, Kazuo Sakamoto, Tomomi Nagayama, Hirotake Yokoyama, Tsukasa Watanabe, Yasushi Mukai, Shunsuke Kawai, Daisuke Yakabe, Hiroshi Mannoji, Kazuhiro Nagaoka, Atsushi Tanaka, Mitsutaka Yamamoto, Kiyohiro Ogawa, Takeshi Mikami, Shujiro Inoue, Susumu Takase, Kei Inoue, Kazuya Hosokawa, Koji Todaka, Hiroyuki Tsutsui, Kohtaro Abe

**Affiliations:** Department of Cardiovascular Medicine, Faculty of Medical Sciences, Kyushu University, Fukuoka, Japan; Centre for Clinical and Translational Research of Kyushu University Hospital, Fukuoka, Japan; Institute for Medical Engineering & Science, Massachusetts Institute of Technology, Cambridge, USA; School of Health Sciences, International University of Health and Welfare, Fukuoka, Japan; Department of Cardiovascular Medicine/Coronary Care Unit, Kyushu University Hospital, 3-1-1, Maidashi, Higashi-ku, Fukuoka 812-8582, Japan; Department of Cardiovascular Medicine, Faculty of Medical Sciences, Kyushu University, Fukuoka, Japan; Centre for Clinical and Translational Research of Kyushu University Hospital, Fukuoka, Japan; Department of Cardiovascular Medicine, Graduate School of Medical Sciences, Kyushu University, Fukuoka, Japan; Department of Cardiovascular Medicine, Graduate School of Medical Sciences, Kyushu University, Fukuoka, Japan; Department of Cardiology, Japanese Red Cross Fukuoka Hospital, Fukuoka, Japan; Department of Cardiology, Japanese Red Cross Fukuoka Hospital, Fukuoka, Japan; Department of Cardiovascular Internal Medicine, Clinical Research Institute, National Hospital Organization Kyushu Medical Center, Fukuoka, Japan; Department of Cardiovascular Medicine, Hamanomachi Hospital, Fukuoka, Japan; Department of Cardiovascular Medicine, St.Mary's Hospital, Fukuoka, Japan; Division of Cardiology, Cardiovascular and Aortic Center, Saiseikai Fukuoka General Hospital, Fukuoka, Japan; Department of Cardiovascular Medicine, Harasanshin Hospital, Fukuoka, Japan; Department of Cardiology, Fukuoka City Hospital, Fukuoka, Japan; Department of Cardiology, Munakata Suikokai General Hospital, Fukuoka, Japan; Department of Cardiology, Aso Iizuka Hospital, Iizuka, Japan; Department of Cardiovascular Medicine, Kyushu University Hospital, Fukuoka, Japan; SUSMED, Inc., Tokyo, Japan; Department of Cardiovascular Medicine, Faculty of Medical Sciences, Kyushu University, Fukuoka, Japan; Centre for Clinical and Translational Research of Kyushu University Hospital, Fukuoka, Japan; Centre for Clinical and Translational Research of Kyushu University Hospital, Fukuoka, Japan; School of Health Sciences, International University of Health and Welfare, Fukuoka, Japan; Department of Cardiovascular Medicine, Graduate School of Medical Sciences, Kyushu University, Fukuoka, Japan

**Keywords:** Atrial fibrillation, Catheter ablation, Artificial intelligence, Machine learning, Voltage map, Electrophysiological map

## Abstract

**Aims:**

Pulmonary vein isolation (PVI) has been established as the standard catheter ablation (CA) strategy for atrial fibrillation (AF). However, approximately 20–40% of patients experience recurrence after CA. Although three-dimensional (3D) maps generated during CA provide valuable electrophysiological information, they may not be fully utilized in clinical decision-making. To develop an artificial intelligence (AI) model that analyses 3D voltage maps and long-term AF recurrence to guide best practices in CA for AF.

**Methods and results:**

A dedicated multicentre registry recording detailed CA data for AF and recurrence was used to develop the AI model. The model was designed to evaluate the completion of PVI and ablation beyond-PVI (be-PVI), considering future AF recurrence with the need for additional PVI and be-PVI interventions. The AI model was trained and validated via fivefold cross-validation with 1268 maps. It effectively stratified cases for predicting 1-year AF recurrence after CA (*P* < 0.001) and identified those likely to benefit from additional ablations (PVI: *P* = 0.032, be-PVI: *P* < 0.001, and a combination of PVI and be-PVI: *P* < 0.001).

**Conclusion:**

The developed AI model predicts AF recurrence based on the completion of PVI and be-PVI and accurately identifies patients who may require further intervention. AI analysis of intraoperative 3D maps could guide optimal CA strategy planning, considering long-term AF recurrence.

## Graphical Abstract

**Graphical Abstract:**
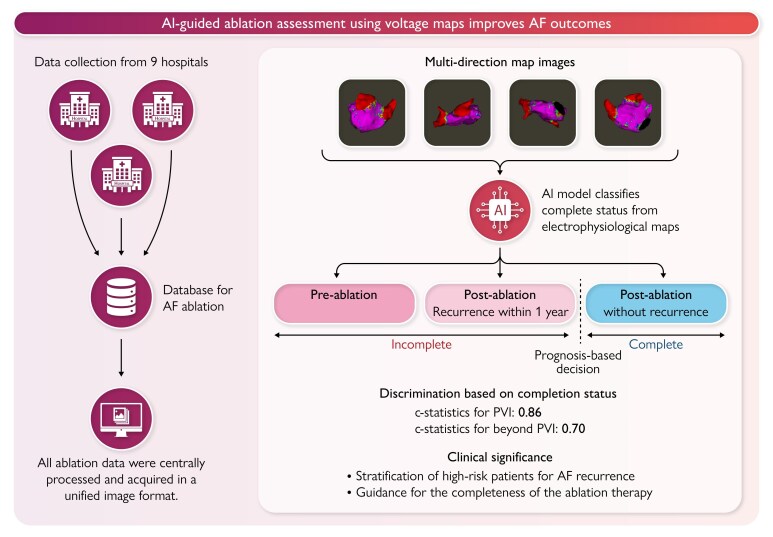

## Introduction

Atrial fibrillation (AF) has become a common cardiovascular disease with a global prevalence that has increased substantially from 33.5 million in 2010 to 59 million in 2019.^1^ The disorder significantly limits the quality of life of patients and worsens the prognosis of patients with heart failure, stroke, and dementia.^2–4^ Numerous studies have demonstrated that catheter ablation (CA) for AF can mitigate these risks,^5,6^ which has led to a worldwide surge in the number of CA procedures performed.

Pulmonary vein isolation (PVI) is the cornerstone strategy of CA for treating AF; however, a significant challenge remains, with 20–40% of patients experiencing recurrence after PVI.^7^ Recurrence mechanisms include pulmonary vein (PV) reconduction and arrhythmogenic substrates beyond the PVs, such as non-PV triggers and atrial tachycardia (AT).^8^ Although the procedural simplicity and durability of PVI have improved drastically with advanced CA technologies, including pulsed-field ablation, the ablation beyond-PVI (be-PVI) strategy remains underdeveloped.^9,10^ This challenge stems from the high interpatient variability of AF substrates, necessitating patient-tailored CA strategies.

Three-dimensional (3D) maps, including rich electrophysiological information, have advanced significantly and become essential for tailored CA for each AF patient.^11^ In particular, voltage maps depicting local electrogram amplitudes can visualize the atrial arrhythmogenic substrate by differentiating between healthy and diseased myocardium; however, human visual interpretation alone is insufficient to realize the full potential of voltage maps.^9,10^

We hypothesized that an artificial intelligence (AI)-driven analysis of intraoperative voltage maps could optimize CA strategy by incorporating long-term outcome data. To verify our hypothesis, we developed an AI model that analyses multidirectional left atrial (LA) voltage maps to evaluate ablation completeness for both PVI and be-PVI, trained on long-term recurrence data from a dedicated multicentre registry.

## Methods

### Data and participants

We established a registry to collect data between April 2016 and March 2023 at nine centres in Japan. The study retrospectively enrolled patients with AF and related arrhythmias aged ≥18 years who underwent CA. Patients with complicated congenital heart disease or ablation for atrioventricular nodal re-entrant tachycardia, atrioventricular re-entrant tachycardia, or common atrial flutter were excluded. However, patients with a history of CA for AF were included. Patient demographics, clinical background, laboratory data, physiological tests, and CA records, including 3D map images, were collected. Data on complications and AF recurrence for up to 2 years after CA were also collected. AF recurrence was based on its recording on a surface ECG or an episode lasting ≥30 s, as documented by a cardiac implantable electronic device. Given the exploratory nature of this proof-of-concept study and the data-intensive requirements of AI model development, we aimed to enrol as many patients as possible rather than performing a formal sample size calculation. This study was approved by the Institutional Review Board of Kyushu University and was conducted in accordance with the Declaration of Helsinki. As a multicentre registry, each participating institution followed its own institutional protocol for intra-procedural lesion assessment. No patient or public involvement was included in this study because of its retrospective observational design. In addition, the study followed the EHRA AI checklist for reporting AI-based prediction model development and validation (see Supplementary material online, *Table S4*).^12^

### Data preparation

The bipolar voltage maps of the entire LA, obtained during CA, were used as input data in the present study because they represent the most standardized electrophysiological data acquired during CA. Right atrial (RA) maps or partial LA maps were excluded. The original data were anonymized and centrally collected. The projection settings on the CARTO 3 system (Biosense Webster Inc., Irvine, CA, USA) were standardized to ensure consistency across institutions. The LA appendage was digitally removed from the images using the CARTO 3 system because it physically overlapped with the LA. The processed 3D data were output as non-transparent two-dimensional (2D) images from four directions: Anterior–posterior (AP), posterior–anterior (PA), inferior (INF), and superior (SUP). All the images from the four directions were saved as 256 × 256-pixel images in a unified RGB format.

### Voltage map categorization and outcome definition

**Figure 1:**
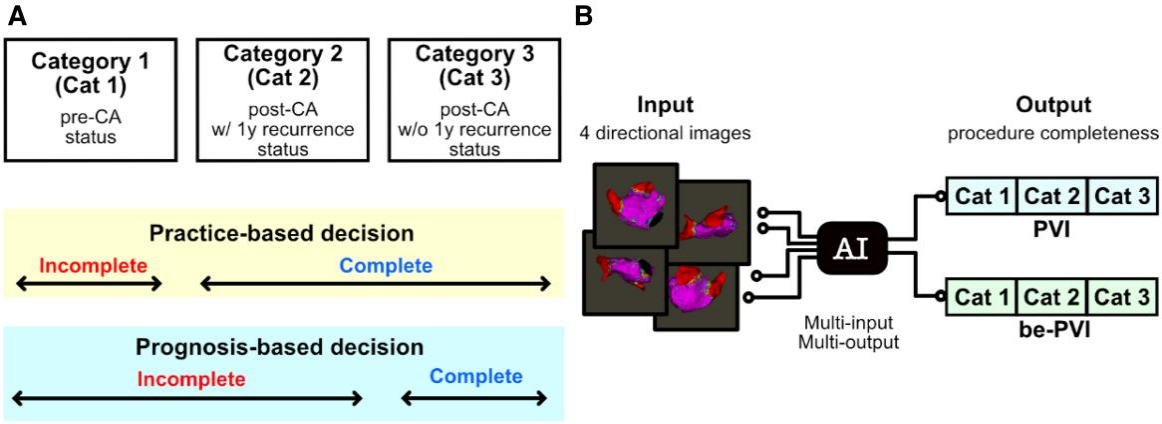
Classification framework for voltage map analysis and AI model architecture. (*A*) Three categories of model outputs based on map acquisition timing and 1-year outcomes (see main text for detailed category definitions). ‘Practice-based decision’ differentiates Category 1 from Categories 2 and 3, evaluating ablation completion without considering long-term recurrence. ‘Prognosis-based decision’ differentiates Categories 1 and 2 from Category 3, evaluating ablation completion while considering future recurrence. (*B*) Architecture of the AI model. Three-dimensional voltage map images from four directions were used as inputs and processed using a lightweight deep learning model based on ConvMixer. The output layer predicts the probabilities for the three categories for both pulmonary vein isolation (PVI) and beyond-PVI (be-PVI) regarding treatment completion.

*Figure 1A* illustrates how the AI model categorizes voltage maps into three groups based on map acquisition timing and 1-year outcomes. The categorization is performed independently for PVI and be-PVI. Maps in Category 1 represent pre-intervention (i.e. requiring intervention) status, acquired before PVI or be-PVI was performed. Maps in Categories 2 and 3 represent post-intervention (i.e. considered complete during the session) status, with no further PVI or be-PVI performed after map acquisition during the same session. Category 2 includes cases with AF recurrence within 1 year, requiring ablation of the same target. Cases that did not require ablation of the same target for at least 1 year were grouped into Category 3.

All the voltage maps were classified into one of the three categories. The AI model in this study directly predicted the probability distributions of these three categories, which represent the ablation completion statuses of PVI and be-PVI. If the patient could no longer be followed up within 1 year or if the target of the repeat ablation (PVI or be-PVI) was unknown for recurrence cases, the data were censored. AF recurrence was defined as overall AF recurrence, irrespective of PVI and be-PVI; the cases were censored if patient follow-up ceased within 1 year.

Regarding class distribution, among the voltage maps with observed outcomes for PVI (*n* = 988, non-censored), Category 1 (pre-intervention), Category 2 (post-intervention with recurrence), and Category 3 (post-intervention without recurrence) accounted for 56.2%, 3.1%, and 40.7%, respectively. For be-PVI (*n* = 897, non-censored), the corresponding proportions were 29.4%, 7.8%, and 62.8%.

Differentiating Category 1 from Categories 2 and 3 implies that the AI determines whether ablation is complete for PVI or be-PVI based on the voltage map alone, mimicking the actual operators’ decision (practice-based decision). Differentiating Categories 1 and 2 from Category 3 implies that the AI determines whether ablation is complete, considering long-term AF recurrence (prognosis-based decision). This study primarily focused on the ‘prognosis-based decision’, as it has greater clinical value.

### Analytical methods

Given the limited dataset size for this proof-of-concept study, the model was evaluated using fivefold cross-validation; in each fold, approximately 80% of patients were allocated to the training set and 20% to the testing set, with no overlap of patients between the two sets. The training set was used for model development, and the testing set was used exclusively for performance evaluation. The final results were obtained by pooling predictions across all five folds. The AI model comprised a multi-input multi-output architecture and was trained on voltage maps from four directions to classify PVI and be-PVI interventions into three categories (*Figure 1B*). The architecture was designed with a lightweight convolutional neural network (CNN) based on ConvMixer^13^ to achieve satisfactory performance with only a few parameters. To avoid overfitting, hyperparameters were set conservatively using a minimal ConvMixer architecture (depth = 4, filters = 16, patch size = 4). The model performed multiclass classification using the censored data. Loss functions comprised ranking loss and log-likelihood loss based on DeepHit,^14^ which adapts time-to-event analysis for deep learning frameworks. For data processing, the image data were scaled from zero to one; only random left‒right flips were used for data augmentation to maintain the positional relationship across the four images.

The model performance was evaluated by considering the time-dependent c-statistic and receiver operating characteristic curve. Long-term outcomes were compared by applying Kaplan–Meier curves and log-rank tests to the pooled results of each cross-validation. The significance level was set to 0.05. Given the proof-of-concept nature of this study and the limited number of cases, the heterogeneity in model performance, such as the clustering of institutions or the fairness of the model, was not examined. TensorFlow 2.15 was used for the analysis.

## Results

**Figure 2:**
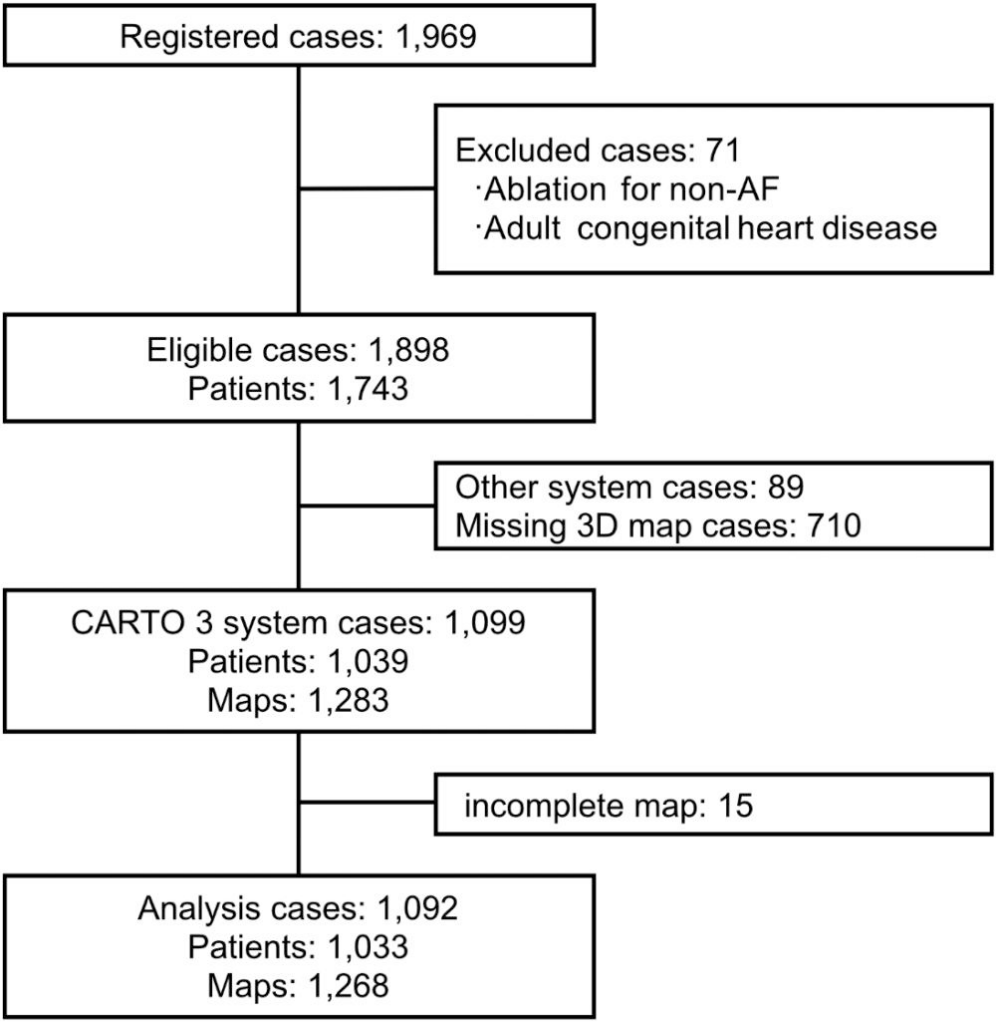
Patient selection flow diagram for the multicentre AI study. The data were analysed via fivefold cross-validation based on the patients. AF: atrial fibrillation.

As shown in *Figure 2*, 1969 cases were enrolled, excluding ineligible cases and those without ablation data in the CARTO 3 system. In addition, cases with insufficient voltage maps that did not include the entire LA chamber were excluded. Finally, 1092 cases with 1268 voltage maps were analysed. *Table 1* shows the overall characteristics of the patients. The median age at CA was 71 years, with paroxysmal AF present in 44.1% of patients. Most of the procedures were performed during the first session (70.1%). The median LA diameter measured by echocardiography was 42.0 mm, and this cohort included patients with LA enlargement. The median left ventricular ejection fraction was 63.8%, and 29.3% of patients had underlying organic heart disease. Furthermore, 61.0% of the patients were prescribed antiarrhythmic medications at discharge after CA. The AF recurrence rate at 1 year in this population was 23.3% [95% confidence interval (CI): 20.7–26.1].

**Table 1:** Patient characteristics

Features	Ablation cases, *N* = 1092
Number of patients	1033
Number of maps	1268
Age [IQR]	71 [63–77]
Female, *n* (%)	350 (32.1)
BMI, kg/m^2^ [IQR]	23.7 [21.3–26.5]
PAF, *n* (%)	482 (44.1)
Ablation session	
1st session, *n* (%)	766 (70.1)
2nd session, *n* (%)	264 (24.2)
3rd + session, *n* (%)	62 (5.7)
Organic heart disease, *n* (%)	320 (29.3)
Heart failure, *n* (%)	303 (27.7)
Hypertension, *n* (%)	672 (61.5)
Diabetes mellitus, *n* (%)	215 (19.7)
History of stroke or TIA, *n* (%)	133 (12.2)
Left atrial diameter, mm [IQR]	42.0 [37.0–46.0]
Left ventricular ejection fraction, % [IQR]	63.8 [56.9–69.4]
Antiarrhythmic drugs at discharge, (%)	666 (61.0)
1-year recurrence rate, % [95% CI]	23.3 [20.7–26.1]

Values are represented as *N* (%), median [interquartile range (IQR)], or mean [95% confidence interval (CI)]. BMI, body mass index; PAF, paroxysmal atrial fibrillation; TIA, transient ischaemic attack.

### AI performance under the ‘prognosis-based decision’

**Figure 3:**
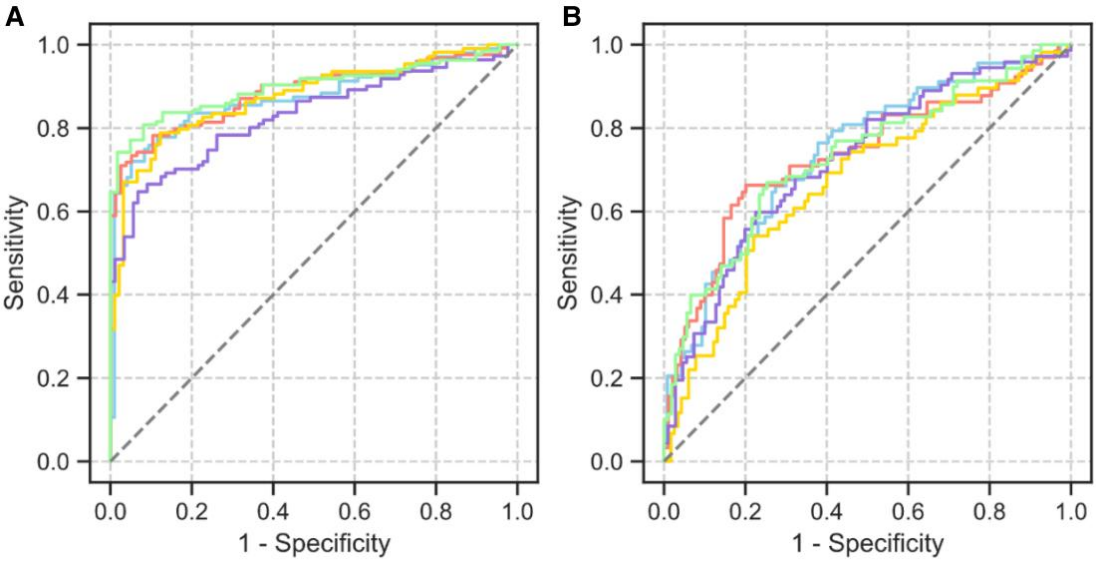
Discriminative performance of the ‘prognosis-based decision’. Evaluation of the discriminative performance of the ‘prognosis-based decision’ with fivefold cross-validation. (A) Represents the time-dependent receiver operating characteristic curves of PVI and (B) Represents those of be-PVI.

For the ‘prognosis-based decision’, which determined ablation completion status based on future AF recurrence, the mean and standard deviation of the time-dependent c-statistics for PVI and be-PVI were 0.86 ± 0.03 and 0.70 ± 0.02, respectively (*Figure 3*), with training set values of 0.88 ± 0.01 and 0.71 ± 0.01. For the ‘practice-based decision’, which determined ablation completion status without considering the potential for future recurrence, the time-dependent c-statistics (mean ± SD) were 0.89 ± 0.03 for PVI and 0.72 ± 0.02 for be-PVI (see Supplementary material online, *Figure S1*). The AI visualization (see Supplementary material online, *Figure S2*) revealed that the model focused on different areas for each prediction task (PVI and be-PVI). For predicting PVI completion, the model focused on the PV area, whereas for predicting be-PVI completion, the model focused on the PV-atrial border (PV antrum) and the low-voltage border region.

### Prediction of long-term AF recurrence by the ‘prognosis-based decision’

**Figure 4:**
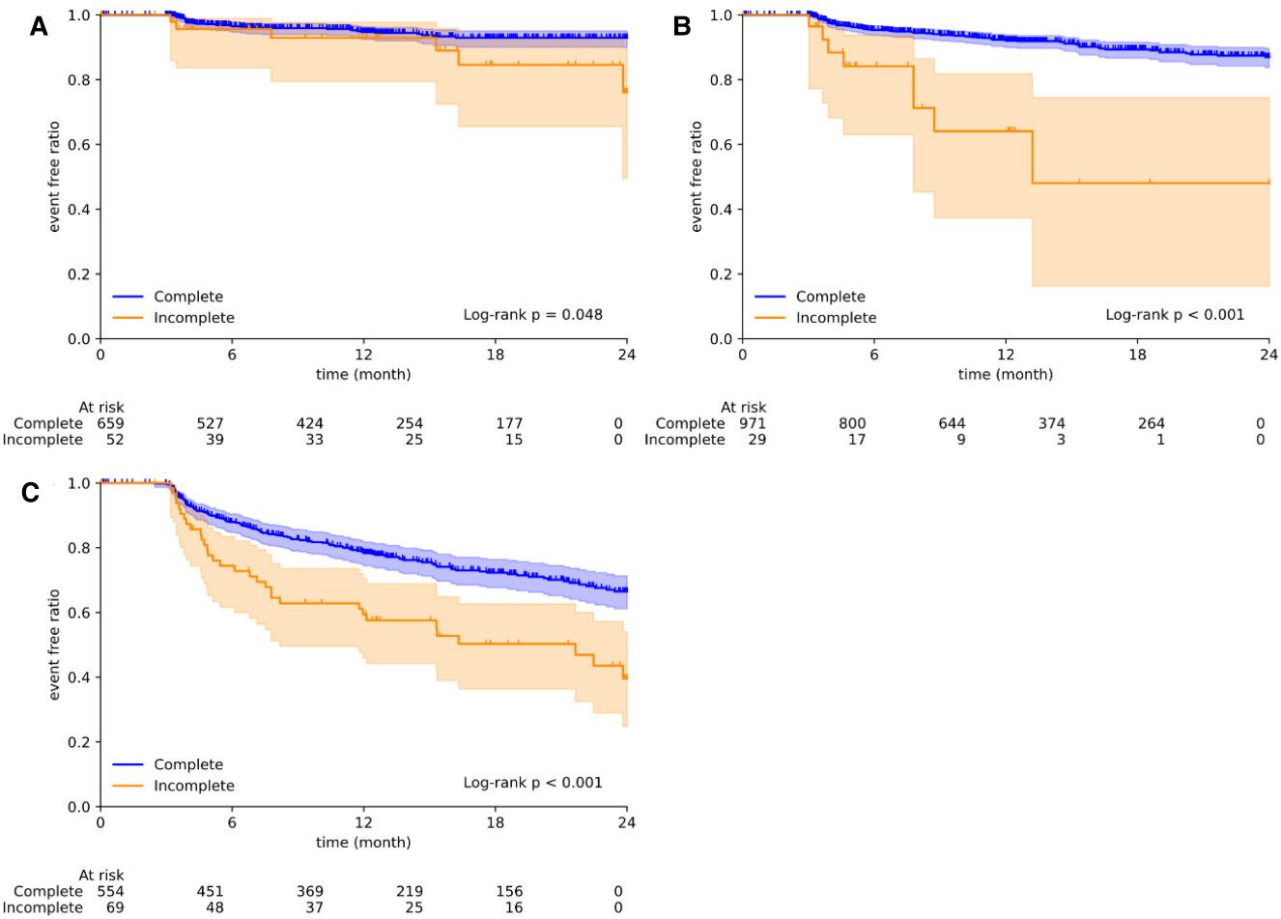
Kaplan–Meier curves for AF recurrence risk stratified by the ‘prognosis-based decision’. The AI performance for the ‘prognosis-based decision’ is evaluated using post-ablation voltage maps. Each panel shows the prognosis based on the intervention type: AF recurrence requiring redo-PVI (*A*), redo-beyond-PVI (*B*), or any AF recurrence regardless of redo strategy (*C*). ‘Complete’ and ‘Incomplete’ indicate AI predictions of ablation status based on electrophysiological findings, with consideration of subsequent clinical outcomes.

*Figure 4* shows the long-term AF recurrence stratified by the ‘prognosis-based decision’ for redo-PVI (A), redo-be-PVI (B), and AF recurrence of any cause (C) in patients whose voltage maps were acquired at the end of the CAs. The cases classified by the AI model as having an incomplete status were highly prone to AF recurrence in the long term (*P* < 0.05 in all analyses). The risk of redo-PVI or be-PVI events was also evaluated via multivariate Cox analysis; the analysis revealed that AI classification as incomplete status remained a strong prognostic factor even after adjustment for clinical covariates (Supplementary Table 1; HR [95% CI] = 1.95 [1.31–2.89]; *P* < 0.01).

Stratification by the ‘practice-based decision’ (see Supplementary material online, *Figure S3*), which determines either complete or incomplete status without considering the potential for future recurrence, was less effective than stratification by the ‘prognosis-based decision’. With respect to redo-be-PVI, few cases were classified as ‘high risk’ by the model, and there were no statistically significant differences in redo-PVI or AF recurrence.

### Impact of additional ablation on AF recurrence

**Figure 5:**
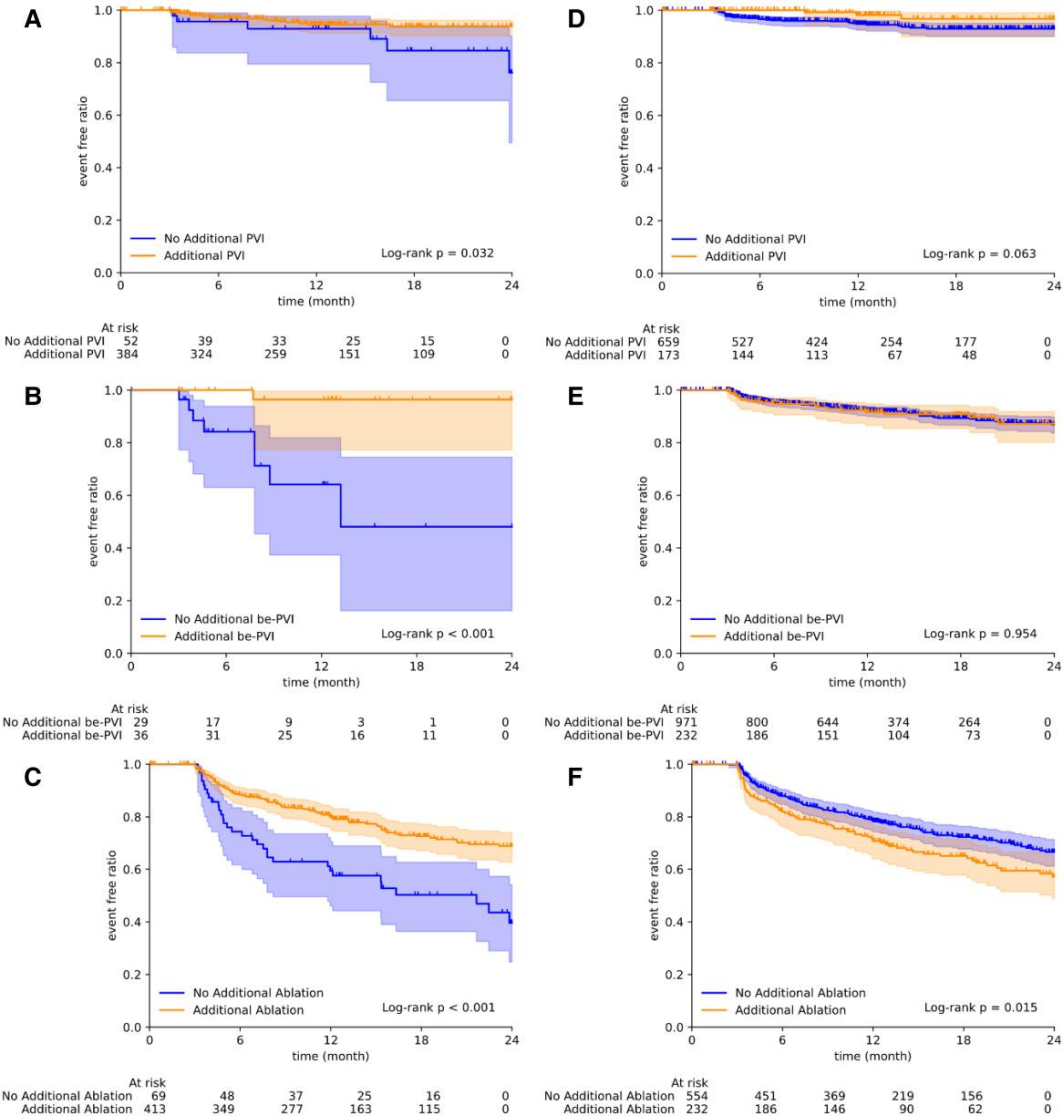
AI-based assessment of impact of additional ablation on AF recurrence. The impact of additional ablation on AF recurrence was compared in cases classified as ‘incomplete’ by the prognosis-based decision, stratified by ablation type: PVI alone (*A*), beyond-PVI (be-PVI) alone (*B*), and either PVI and/or be-PVI (*C*). Within each type, cases were compared between those without additional ablation after mapping (No Additional *) and those with additional ablation after mapping (Additional *). Similarly, the impact of additional ablation was compared in cases classified as ‘complete’ by the prognosis-based decision, stratified by ablation strategy: PVI (*D*), be-PVI (*E*), and either PVI or be-PVI (F).

The effect of additional ablation on AF recurrence in patients stratified by the AI model was analysed via both intraoperative and post-ablation voltage maps. All voltage maps were divided into two subgroups: those assessed by the AI model as incomplete or complete for PVI, be-PVI, and AF recurrence. In the subgroups assessed as incomplete for PVI, be-PVI, and AF recurrence (*Figures 5A–C*), the incidence of AF recurrence was significantly lower in patients who underwent additional ablation in the same session than in those who did not undergo additional ablation. The trend was similar in the adjusted Cox analysis for AF recurrence (see Supplementary material online, *Table S2*, hazard ratio [95% CI]: 0.58 [0.34–0.99], and *P* = 0.05).

By contrast, no significant difference in redo-PVI events in patients classified as having a complete status for PVI was observed regardless of whether the patient received subsequent PVI in the same session (*Figure 5D*). Similarly, for redo events of be-PVI (*Figure 5E*), no significant differences were observed with or without subsequent ablation. Although AF recurrence events appeared more frequent in the group that received additional ablation (*Figure 5F*), this association was not significant after multivariate adjustment (see Supplementary material online, *Table S3*; hazard ratio [95% CI]: 0.95 [0.67–1.35], *P* = 0.77).

## Discussion

3D electroanatomic maps, which convert intracardiac electrophysiological observations into voltage and activation maps, have become integral to contemporary CA strategies.^11,15^ These maps provide intuitive and valuable insights into arrhythmogenic substrates in patients with AF; however, human visual interpretation alone is insufficient to realize their full potential. To explore whether AI could leverage this rich electrophysiological information, we established a dedicated registry and conducted this proof-of-concept study. The proposed model assesses ablation completeness for both PVI and be-PVI while considering long-term recurrence, providing insights into whether additional ablation may prevent AF recurrence.

### AI modelling for predicting AF recurrence from voltage maps

Developing an AI model to analyse voltage maps presents unique challenges, including inter-institutional variability in image acquisition and the complexity of 3D spatial data. We addressed these challenges through careful data standardization and model architecture design.

As imaging conditions depend on the operator and institution, standardizing data acquisition settings from the CARTO system and the 3D reconstructed image configuration minimized inter-institutional variability. Owing to system constraints and computing limitations that impede the direct use of 3D map data, images from four directions (AP, PA, INF, and SUP) were aggregated and fed into the AI model. Additionally, the model was designed to predict the ablation completion status, using Categories 1–3 for both PVI and be-PVI. The three completion categories for PVI and be-PVI were defined as follows: Category 1, representing the pre-CA status; Category 2, indicating a post-CA status with high AF recurrence risk; and Category 3, representing a post-CA status with low AF recurrence risk. Owing to the nature of ordinal categories and the potential for censoring, the DeepHit framework was used for modelling.^14^ This framework can predict discrete time-to-event outcomes. As the original DeepHit model was designed for tabular data, the input and intermediate layers were replaced with a CNN-based image classification architecture for time-to-event prediction from the images. The backbone architecture comprised a ConvMixer-based CNN^13^ with high performance and fewer parameters than other foundation models. Preliminary experiments were also conducted using transfer learning from pre-trained models (ResNet and EfficientNet). However, these approaches yielded suboptimal performance. Several factors might have contributed to this limitation. First, the excessive number of parameters in these large models relative to the study’s dataset size (1268 maps) led to overfitting. Second, voltage maps represent highly specialized 3D electrophysiological projections that differ fundamentally from the conventional images used to train foundation models, creating a significant domain gap. Third, the study’s time-to-event prediction task with ordinal categories differed substantially from the standard classification tasks these models were designed for. Given these considerations, we adopted a minimal ConvMixer architecture, which achieved better performance while reducing the risk of overfitting.

### Predictive performance of ‘prognosis-based decision’ and visualization of areas of interest in 3D maps

The model demonstrated good discrimination for PVI completion assessment (c-statistic 0.86), whereas be-PVI prediction was more modest (c-statistic 0.70). The superior performance for PVI classification likely reflects the more defined anatomical targets of PVI, whereas be-PVI encompasses heterogeneous substrates including non-PV triggers, AT, and empirical ablation targets, which vary substantially among patients. This inherent complexity of be-PVI substrates presents a greater challenge not only for operators but also for AI-based prediction. Despite the lower c-statistic for be-PVI, a value of 0.70 still represents clinically meaningful discrimination that can assist operators in identifying patients at higher risk for AF recurrence requiring non-PV intervention. Future studies with larger datasets and more granular substrate characterization may further improve be-PVI prediction.

To better understand the basis for these predictions, we identified regions emphasized by the AI model via saliency maps using the SmoothGrad method.^16^ This method indicates the areas of the input pixels that predominantly influence the model’s prediction. Although quantitative evaluation was challenging, the model emphasized the entire PV area to assess PVI completion. By contrast, for be-PVI, the model focused on the PV-atrial border (PV antrum) and low-voltage areas, suggesting that distinct features were emphasized in the evaluation of PVI and be-PVI. Previous reports have also suggested that low-voltage areas serve as potential sources of arrhythmias,^17–19^ and the areas of interest were consistent with these known pathophysiological findings. While these saliency maps (see Supplementary material online, *Figure S2*) offer anatomical insight into the model’s decision-making, the current architecture provides global risk stratification rather than region-specific ablation recommendations. Toward clinical implementation, we envision a workflow in which the model analyses the voltage map at key procedural timepoints (e.g. after initial PVI confirmation or during assessment of be-PVI adequacy) to provide immediate feedback on ablation completeness. Beyond this, extending the model to provide region-specific ablation guidance would be a valuable next evolution of this approach.

### Prognostic value of AI-based risk stratification

The ‘prognosis-based decision’, which incorporates long-term recurrence outcomes, demonstrated superior clinical utility compared with the ‘practice-based decision’. While the ‘practice-based decision’, which mimics operators’ intraoperative judgement, showed limited ability to stratify recurrence risk (see Supplementary material online, *Figure S3*), the ‘prognosis-based decision’ successfully identified patients at high risk for redo-PVI (*P* = 0.032), redo-be-PVI (*P* < 0.001), and overall AF recurrence (*P* < 0.001; *Figure 4*). This difference highlights the added value of incorporating long-term outcome data into AI training, which enables the model to capture prognostic features that may not be apparent during the procedure. The model’s ability to predict redo-be-PVI events (*Figure 4B*) is particularly noteworthy. Identifying patients who will require additional non-PV ablation is clinically challenging, as be-PVI substrates are heterogeneous and often latent at the time of the initial procedure. The AI model’s performance in this regard suggests that voltage maps contain prognostic information about non-PV substrates that is not fully utilized in current practice.

After multivariate adjustment for clinical covariates, AI classification as ‘incomplete status’ remained a strong independent predictor of AF recurrence (HR 1.95, 95% CI 1.31–2.89, *P* < 0.01; Supplementary material online, *Table S1*). This result indicates that the model captures prognostic information from voltage maps beyond conventional clinical risk factors. From a clinical perspective, this risk stratification enables two potential applications. First, intraoperative identification of high-risk patients could prompt operators to consider additional ablation during the same session, potentially reducing the need for repeat procedures. Second, for patients identified as high-risk at the end of the procedure, more intensive post-ablation monitoring and earlier antiarrhythmic medication adjustment may be warranted. This represents a shift from uniform post-ablation management to a personalized, risk-stratified approach.

### Potential of AI-guided ablation strategies

We also evaluated the model’s performance as an AI guide for ablation completion. All the cases were divided into two subgroups based on AI model predictions: one evaluated as incomplete ablation and the other as complete ablation. When the AI model deemed the ablation incomplete (*Figures 5A–C*), additional ablation significantly improved the outcomes. Conversely, additional ablation did not significantly improve the prognosis in the subgroup that the AI model deemed complete (*Figures 5D–F*). This finding requires careful interpretation and has several potential explanations. One optimistic interpretation is that, when the AI model assesses ablation as complete, performing additional ablation may cause unnecessary tissue damage, thereby creating new arrhythmogenic substrates or disrupting the existing protective anatomical barriers. Alternatively, this result may simply reflect selection bias: operators may have chosen to perform additional ablations specifically in patients with higher underlying AF recurrence risk. However, multivariate Cox analysis adjusting for clinical covariates showed that additional ablation in the ablation-incomplete group remained beneficial (see Supplementary material online, *Table S2*), supporting the potential clinical utility of AI-guided decision-making.

These findings suggest that this AI model can guide clinicians in two ways: informing clinicians when to perform additional ablation and, just as importantly, when to stop, to avoid causing harm. These findings support the concept of an ‘optimal endpoint’ in CA, in which additional ablation beyond the AI-indicated completion point may be detrimental to patient outcomes. Therefore, the proposed AI model is a potential tool for optimizing CA strategies.

### Novelty and differentiation from existing approaches

Several AI-based models for predicting AF recurrence after CA have been reported, and they predominantly rely on pre-procedural clinical variables,^20–22^ ECG or electrogram signals,^23^ or pre-procedural imaging.^24,25^ Among the studies utilizing intracardiac mapping data, An *et al*.^26^ applied voltage map registration with a multilayer perceptron for AF type and recurrence prediction, and Alhusseini *et al*.^27^ used a CNN to classify intracardiac AF activation patterns. However, the present study differs from existing approaches in several key aspects. First, our model directly analyses intraoperative 3D voltage maps as CNN image inputs for outcome prediction, thereby capturing spatial image features that are not accessible to traditional feature-based models. Second, the model independently evaluates ablation completeness for both PVI and be-PVI, providing a more comprehensive assessment than models that focus solely on overall recurrence prediction. Third, our unique three-category framework incorporating time-to-event analysis enables a ‘prognosis-based decision’ that goes beyond simple binary prediction by distinguishing pre-intervention status, post-intervention with recurrence, and post-intervention without recurrence. Fourth, the analysis of the impact of additional ablation (*Figure 5*) highlights the model’s potential as an intraoperative decision-support tool, which is distinct from purely prognostic models that provide only pre-procedural risk estimates.

### AI application in AF ablation

Although the active search for latent AF triggers and substrates, including non-PV origins and ATs, has been useful for suppressing AF recurrence,^28,29^ excessive AF induction can lead to prolonged CA times and unnecessary ablations targeting nonclinical arrhythmias,^30^ resulting in increased complication risk.^31^

Our AI model offers a potential solution to this dilemma by providing an objective, prognosis-based assessment of ablation completeness. Rather than relying solely on acute procedural endpoints or empirical induction protocols, the model integrates voltage map patterns with long-term outcome data to guide intraoperative decision-making, identifying when additional ablation may be beneficial and when it may not be necessary. Nevertheless, careful implementation is essential. Previous studies have shown that while AI can assist novice physicians, biased AI models may paradoxically reduce the diagnostic accuracy of highly skilled practitioners.^32,33^ The interaction between AI guidance and operator expertise must therefore be carefully considered. Clinical implementation will require comprehensive validation, including prospective data collection, algorithm refinement, real-world performance assessment across diverse settings, and evaluation of AI–operator interactions to ensure both patient safety and clinical efficacy.

### Limitations

This study has several important limitations. First, its retrospective, single-country design using the CARTO 3 system exclusively across nine experienced Japanese centres precludes definitive causal inferences and may limit generalizability to other populations, practice settings, and mapping technologies. Additionally, potential health disparities across sociodemographic groups were not specifically examined. Prospective randomized trials involving diverse patient populations and healthcare systems are essential to establish true clinical utility and safety. Second, distinguishing iatrogenic recurrence attributable to incomplete lesion durability or excessive ablation from recurrence driven by intrinsic arrhythmogenic substrates remains a fundamental clinical challenge that cannot be resolved even with standardized protocols, as these mechanisms are clinically inseparable. Third, although the study cohort included patients with heart failure and organic heart disease, patients with severely reduced ejection fraction and markedly enlarged atria were underrepresented in the training data. Dedicated evaluation with larger cohorts enriched with patients exhibiting advanced atrial remodelling is warranted. Fourth, we limited our analysis to the LA because it is uncommon to draw complete RA maps in CA for AF. However, RA-originating AF is not negligible, and a comprehensive AI model that includes RA data must be developed for future clinical applications.^34^ Finally, the current model relies exclusively on bipolar voltage maps converted to 2D images, resulting in inherent information loss and limiting the electrophysiological features available for analysis. Although our four-directional imaging strategy captures major anatomical features, important spatial relationships and voltage gradients in the depth dimension are not preserved. Future studies should explore both the integration of additional electrogram characteristics, including fractionation, signal duration, conduction velocity, and unipolar voltage data, and the application of direct 3D CNNs or native 3D map data analysis to fully utilize the rich spatial information in electroanatomic voltage maps.

## Conclusion

An AI model was developed to enhance best practices in CA for AF by analysing 3D voltage maps and long-term AF recurrence. AI-driven analysis of electroanatomic maps has the potential to facilitate optimal CA strategy planning, thereby guiding operators towards a truly complete CA by accounting for the long-term possibility of requiring additional PVI or be-PVI.

## Supplementary Material

ztag054_Supplementary_Data

## Data Availability

This registry is accessible to collaborators within the project; however, data will not be shared with third parties as restricted by the protocol or research ethics guidelines. These policies apply to both clinical and CARTO data. The analytical code is available from the corresponding author upon reasonable request.

## References

[1] Linz D, Gawalko M, Betz K, Hendriks JM, Lip GYH, Vinter N, et al Atrial fibrillation: epidemiology, screening and digital health. Lancet Reg Health—Eur 2024;37:100786. 38362546 10.1016/j.lanepe.2023.100786 PMC10866942

[2] Fang MC, Go AS, Chang Y, Borowsky LH, Pomernacki NK, Udaltsova N, et al Long-term survival after ischemic stroke in patients with atrial fibrillation. Neurology 2014;82:1033–1037. 24532273 10.1212/WNL.0000000000000248 PMC3962998

[3] Kim D, Yang P-S, Yu HT, Kim T-H, Jang E, Sung J-H, et al Risk of dementia in stroke-free patients diagnosed with atrial fibrillation: data from a population-based cohort. Eur Heart J 2019;40:2313–2323. 31212315 10.1093/eurheartj/ehz386

[4] Diaz J, Martinez F, Calderon JM, Fernandez A, Sauri I, Uso R, et al Incidence and impact of atrial fibrillation in heart failure patients: real-world data in a large community. ESC Heart Fail 2022;9:4230–4239. 36111519 10.1002/ehf2.14124 PMC9773729

[5] Marrouche NF, Brachmann J, Andresen D, Siebels J, Boersma L, Jordaens L, et al Catheter ablation for atrial fibrillation with heart failure. N Engl J Med 2018;378:417–427. 29385358 10.1056/NEJMoa1707855

[6] Benali K, Macle L, Haïssaguerre M, Nattel S, Deyell M, Da Costa A, et al Impact of catheter ablation of atrial fibrillation on disease progression. JACC Clin Electrophysiol 2025;11:421–435. 40010884 10.1016/j.jacep.2024.10.017

[7] Kobza R, Hindricks G, Tanner H, Schirdewahn P, Dorszewski A, Piorkowski C, et al Late recurrent arrhythmias after ablation of atrial fibrillation: incidence, mechanisms, and treatment. Heart Rhythm 2004;1:676–683. 15851239 10.1016/j.hrthm.2004.08.009

[8] Erhard N, Mauer T, Ouyang F, Sciacca V, Rillig A, Reissmann B, et al Mechanisms of late arrhythmia recurrence after initially successful pulmonary vein isolation in patients with atrial fibrillation. Pacing Clin Electrophysiol 2023;46:161–168. 36588339 10.1111/pace.14656

[9] Rottner L, Bellmann B, Lin T, Reissmann B, Tönnis T, Schleberger R, et al Catheter ablation of atrial fibrillation: state of the art and future perspectives. Cardiol Ther 2020;9:45–58. 31898209 10.1007/s40119-019-00158-2 PMC7237603

[10] Verma A, Jiang C-y, Betts TR, Chen J, Deisenhofer I, Mantovan R, et al Approaches to catheter ablation for persistent atrial fibrillation. N Engl J Med 2015;372:1812–1822. 25946280 10.1056/NEJMoa1408288

[11] Baykaner T, Narayan SM. Mapping atrial fibrillation to improve ablation outcomes. JAMA Netw Open 2023;6:e2344481–e2344481. 37991767 10.1001/jamanetworkopen.2023.44481 PMC10832580

[12] Svennberg E, Han JK, Caiani EG, Engelhardt S, Ernst S, Friedman P, et al State of the art of artificial intelligence in clinical electrophysiology in 2025: a scientific statement of the European heart rhythm association (EHRA) of the ESC, the heart rhythm society (HRS), and the ESC working group on E-cardiology. EP Europace 2025;27:euaf071. 40163651 10.1093/europace/euaf071 PMC12123071

[13] Trockman A, Kolter JZ. Patches are all you need? https://arxiv.org/abs/2201.09792v1 (2022).

[14] Lee, C., Zame, W. R., Yoon, J. & Van Der Schaar, M. DeepHit: a deep learning approach to survival analysis with competing risks. www.aaai.org. 10.1109/TBME.2019.2909027 30951460

[15] Narayan SM, John RM. Advanced electroanatomic mapping: current and emerging approaches. Curr Treat Options Cardiovasc Med 2024;26:69–91. 41104347 10.1007/s11936-024-01034-6 PMC12525732

[16] Smilkov D, Thorat N, Kim B, Viégas F, Wattenberg M. SmoothGrad: removing noise by adding noise. https://arxiv.org/abs/1706.03825v1 (2017).

[17] Kawai S, Mukai Y, Inoue S, Yakabe D, Nagaoka K, Sakamoto K, et al Non-pulmonary vein triggers of atrial fibrillation are likely to arise from low-voltage areas in the left atrium. Sci Rep 2019;9:12271. 31439861 10.1038/s41598-019-48669-1 PMC6706423

[18] Starek Z, Di Cori A, Betts TR, Clerici G, Gras D, Lyan E, et al Baseline left atrial low-voltage area predicts recurrence after pulmonary vein isolation: WAVE-MAP AF results. EP Europace 2023;25:1–11. 37470443 10.1093/europace/euad194 PMC10410193

[19] Masuda M, Sunaga A, Tanaka N, Watanabe T, Minamiguchi H, Egami Y, et al Low-voltage-area ablation for persistent atrial fibrillation: a randomized controlled trial. Nat Med 2025;31:1661–1667. 40307511 10.1038/s41591-025-03674-y PMC12092238

[20] Saglietto A, Gaita F, Blomstrom-Lundqvist C, Arbelo E, Dagres N, Brugada J, et al AFA-Recur: an ESC EORP AFA-LT registry machine-learning web calculator predicting atrial fibrillation recurrence after ablation. EP Europace 2023;25:92–100. 10.1093/europace/euac145 PMC10103564 36006664

[21] Zhou X, Nakamura K, Sahara N, Takagi T, Toyoda Y, Enomoto Y, et al Deep learning-based recurrence prediction of atrial fibrillation after catheter ablation. Circ J 2022;86:299–308. 34629373 10.1253/circj.CJ-21-0622

[22] Budzianowski J, Kaczmarek-Majer K, Rzeźniczak J, Słomczyński M, Wichrowski F, Hiczkiewicz D, et al Machine learning model for predicting late recurrence of atrial fibrillation after catheter ablation. Sci Rep 2023;13:15213. 37709859 10.1038/s41598-023-42542-y PMC10502018

[23] Tang S, Razeghi O, Kapoor R, Alhusseini MI, Fazal M, Rogers AJ, et al Machine learning-enabled multimodal fusion of intra-atrial and body surface signals in prediction of atrial fibrillation ablation outcomes. Circ Arrhythm Electrophysiol 2022;15:500–509. 10.1161/CIRCEP.122.010850 PMC9972736 35867397

[24] Razeghi O, Kapoor R, Alhusseini MI, Fazal M, Tang S, Roney CH, et al Atrial fibrillation ablation outcome prediction with a machine learning fusion framework incorporating cardiac computed tomography. J Cardiovasc Electrophysiol 2023;34:1164–1174. 36934383 10.1111/jce.15890 PMC10857794

[25] Liu C-M, Chen W-S, Chang S-L, Hsieh Y-C, Hsu Y-H, Chang H-X, et al Use of artificial intelligence and I-Score for prediction of recurrence before catheter ablation of atrial fibrillation. Int J Cardiol 2024;402:131851. 38360099 10.1016/j.ijcard.2024.131851

[26] An Q, McBeth R, Zhou H, Lawlor B, Nguyen D, Jiang S, et al Prediction of type and recurrence of atrial fibrillation after catheter ablation via left atrial electroanatomical voltage mapping registration and multilayer perceptron classification: a retrospective study. Sensors 2022;22:4058. 35684678 10.3390/s22114058 PMC9185445

[27] Alhusseini MI, Abuzaid F, Rogers AJ, Zaman JAB, Baykaner T, Clopton P, et al Machine learning to classify intracardiac electrical patterns during atrial fibrillation: machine learning of atrial fibrillation. Circ Arrhythm Electrophysiol 2020;13:E008160. 32631100 10.1161/CIRCEP.119.008160 PMC7438307

[28] Faustino M, Pizzi C, Agricola T, Xhyheri B, Costa GM, Flacco ME, et al Stepwise ablation approach versus pulmonary vein isolation in patients with paroxysmal atrial fibrillation: randomized controlled trial. Heart Rhythm 2015;12:1907–1915. 26051530 10.1016/j.hrthm.2015.06.009

[29] Huo Y, Gaspar T, Schönbauer R, Wójcik M, Fiedler L, Roithinger FX, et al Low-voltage myocardium-guided ablation trial of persistent atrial fibrillation. NEJM Evid 2022;1:EVIDoa2200141. 38319851 10.1056/EVIDoa2200141

[30] Darma A, Daneschnejad SS, Gaspar T, Huo Y, Wetzel U, Dagres N, et al Role of inducibility and its dynamic change in the outcome of catheter ablation of atrial fibrillation: a single center prospective study. J Cardiovasc Electrophysiol 2020;31:705–711. 31943494 10.1111/jce.14355

[31] Benali K, Khairy P, Hammache N, Petzl A, Da Costa A, Verma A, et al Procedure-related complications of catheter ablation for atrial fibrillation. J Am Coll Cardiol 2023;81:2089–2099. 37225362 10.1016/j.jacc.2023.03.418

[32] Yu F, Moehring A, Banerjee O, Salz T, Agarwal N, Rajpurkar P. Heterogeneity and predictors of the effects of AI assistance on radiologists. Nat Med 2024;30:837–849. 38504016 10.1038/s41591-024-02850-w PMC10957478

[33] Jabbour S, Fouhey D, Shepard S, Valley TS, Kazerooni EA, Banovic N, et al Measuring the impact of AI in the diagnosis of hospitalized patients: a randomized clinical vignette survey study. JAMA 2023;330:2275–2284. 38112814 10.1001/jama.2023.22295 PMC10731487

[34] Santangeli P, Marchlinski FE. Techniques for the provocation, localization, and ablation of non–pulmonary vein triggers for atrial fibrillation. Heart Rhythm 2017;14:1087–1096. 28259694 10.1016/j.hrthm.2017.02.030

