# Supplementary material for: AI-driven voltage map analysis for optimizing catheter ablation strategy in atrial fibrillation: a proof-of-concept study
Source: Eur Heart J Digit Health. 2026 Mar 31;7(4):ztag054. doi: 10.1093/ehjdh/ztag054 (PMC13131983; doi:10.1093/ehjdh/ztag054)
Supplement: ztag054_Supplementary_Data [file ztag054_supplementary_data.zip › Supplementary_Table4_EHRA_Checklist.docx]

# Supplementary Table 4. EHRA AI Checklist Mapping

*Systematic mapping of the EHRA AI Checklist for reporting AI studies in clinical electrophysiology (Svennberg et al., Europace 2025;27:euaf071) to the present manuscript.*

| **Item #** | **Category** | **Checklist Item** | **Manuscript Location** |
| --- | --- | --- | --- |
| **TITLE** | | | |
| i | **Title** | Include clear terms to identify the study as using artificial intelligence, machine learning, or other specific terms | Title: “AI-Driven Voltage Map Analysis for Optimising Catheter Ablation Strategy in Atrial Fibrillation: A Proof-of-Concept Study” |
| **INTRODUCTION** | | | |
| 1 | **Intended clinical use** | Clearly describe the intended use and where in clinical workflow the model can be used and the objective of the study | Introduction, paragraphs 3–4: AI-driven analysis of intraoperative voltage maps to optimise CA strategy incorporating long-term outcome data |
| 2 | **Clinical benefit** | Added benefit of AI compared with standard clinical care (gold standard) | Introduction, paragraph 3: Human visual interpretation alone is insufficient; Discussion, "Potential of AI-guided ablation strategies" |
| **METHODS** | | | |
| 3 | **Data collection** | Describe how data was collected | Methods, "Data and participants": Retrospective registry, April 2016–March 2023, nine centres in Japan |
| 4 | **Source of data** | Describe the study design or source of input data and how it was acquired | Methods, "Data and participants": Multicentre retrospective registry with centrally collected anonymised data |
| 5 | **Development dataset** | Describe the data set | Methods, "Analytical methods": Fivefold cross-validation with 80% training / 20% testing split per fold |
| 6 | **Participants** | Describe the participants in the data sets, including eligibility criteria (inclusion and exclusion criteria). | Methods, "Data and participants": Inclusion/exclusion criteria described; Results, Figure 2 (flow diagram) and Table 1 (demographics) |
| 7 | **Comparator** | Provide clear definition of how the gold standard was collected. Clearly describe the gold standard and ground truth including limitations. | Methods, "Voltage map categorisation and outcome definition": AF recurrence was based on its recording on a surface ECG or an episode lasting ≥30 s, as documented by a cardiac implantable electronic device. |
| 8 | **Testing dataset** | Describe the testing data set, in particular defining the data set split. | Methods, "Analytical methods": 20% patient-level testing set per fold, no overlap between folds |
| 9 | **Sample size** | Explain how the study size was arrived at. | Methods, "Data and participants": Proof-of-concept; enrolled as many patients as possible. Results: 1,092 cases, 1,268 maps. Class distribution provided |
| 10 | **Outcome** | Clearly define standardized and reproducible outcome of clinical relevance. | Methods, "Voltage map categorisation and outcome definition": Three-category classification for PVI and be-PVI with 1-year recurrence endpoint |
| 11 | **Data type** | Clearly describe the data type for the study, including pre-processing | Methods, "Data preparation": Bipolar voltage maps from CARTO 3, 256×256 RGB images from four directions. Bipolar-only rationale clarified |
| 12 | **Data preparation** | Input data handling, data augmentation and selection prior to analysis by the AI system, application of techniques to prevent data leakage. | Methods, "Data preparation" and "Analytical methods": Standardised projection settings, LAA removal, patient-level split to prevent data leakage, random L–R flips |
| 13 | **Balanced groups** | Clearly state how/if groups were balanced | Methods, "Analytical methods": Class distribution reported (PVI: 56.2%, 3.1%, and 40.7%; be-PVI: 29.4%, 7.8%, and 62.8%). |
| 14 | **Data issues** | Describe how handling of data of poor quality/noise/missing data was performed | Methods, "Data preparation" and Results, Figure 2: Cases with insufficient voltage maps that did not include the entire LA chamber were excluded |
| 15 | **Feature engineering** | If features are used, feature selection should be described including by whom features were extracted. | Methods, "Data preparation": No handcrafted features; CNN learns directly from raw voltage map images in an end-to-end fashion |
| **REGULATORY** | | | |
| 16 | **Legal framework** | Clearly state if the software has been approved by legal authorities, e.g. Certificate of conformity (EU) or FDA approval or other, and add further details, where appropriate (e.g. risk class). | The AI model has not received regulatory approval and is intended for research purposes only |
| 17 | **Explainability** | Is the AI model explainable on the patient level or on a global or local level. | Discussion, "Predictive performance": Saliency maps via SmoothGrad method (Ref. 17); Supplementary Figure 2 |
| 18 | **Ethical approval** | Provide information on ethical approval of the study. | Methods, "Data and participants": Approved by the IRB of Kyushu University; conducted per the Declaration of Helsinki |
| 19 | **Fairness** | Describe inclusion of relevant groups in the dataset | Discussion, "Limitations": Nine geographically distinct centres; acknowledged that health inequalities across sociodemographic groups were not specifically examined |
| **OPEN SCIENCE** | | | |
| 20 | **Data availability** | Is the data available on a public website? Is the code available? | Data sharing statement: Registry accessible to project collaborators; code available from the corresponding author upon reasonable request |
| 21 | **Trial registration** | In case of a trial, clearly state if and where the trial is registered. | Methods, "Data and participants": Retrospective observational study; prospective trial registration was not applicable |
| **RESULTS** | | | |
| 22 | **Participants** | Baseline demographics (internal and external validation data). | Results, Table 1: Complete baseline demographics for 1,092 ablation cases; Figure 2: Patient flow diagram |
| 23 | **Training performance** | Provide results from the training data set | Results, 'AI performance under the prognosis-based decision': Training set c-statistics reported alongside test set values (PVI: 0.88 ± 0.01; be-PVI: 0.71 ± 0.01). |
| 24 | **Internal validation** | The results from the testing data set | Results, Figure 3: Time-dependent c-statistics (PVI: 0.86±0.03; be-PVI: 0.70±0.02); Figure 4: Kaplan–Meier curves |
| 25 | **External validation** | The results from the external validation data set | Not performed. Acknowledged in Limitations as requiring international validation across diverse populations |
| 26 | **Model performance** | Choose appropriate metric selection for reporting | Results: Time-dependent c-statistics, ROC curves (Figure 3), Kaplan–Meier analysis (Figure 4), multivariate Cox regression (Supplementary Tables 1–3) |
| 27 | **Performance errors** | Analysis of performance errors and how they were identified | Misclassification patterns were assessed through fold-level variability in c-statistics and survival analysis |
| 28 | **Comparison with conventional methods** | What did the model add? | Direct comparison with conventional statistical models is not applicable, as the model input consists of voltage map images that cannot be directly analysed in their native image format by traditional statistical methods. However, multivariate Cox regression demonstrated that AI classification remained an independent predictor after adjustment for clinical covariates. |
| 29 | **Generalizability** | Discuss the level of generalizability of the results obtained. | Discussion, "Limitations": Japanese-only cohort, CARTO 3 system only, underrepresentation of severe HFrEF; international validation needed |
| **CONCLUSION** | | | |
| ii | **Conclusion** | Is the conclusion supported by the dataset? | Supported. Findings framed as proof-of-concept requiring prospective validation. |

*EHRA = European Heart Rhythm Association; AI = artificial intelligence; EP = electrophysiology; PVI = pulmonary vein isolation; be-PVI = beyond PVI; CA = catheter ablation; AF = atrial fibrillation; CNN = convolutional neural network; IRB = Institutional Review Board; CIED = cardiac implantable electronic device; HFrEF = heart failure with reduced ejection fraction.*
