# Supplementary material for: AI-driven voltage map analysis for optimizing catheter ablation strategy in atrial fibrillation: a proof-of-concept study
Source: Eur Heart J Digit Health. 2026 Mar 31;7(4):ztag054. doi: 10.1093/ehjdh/ztag054 (PMC13131983; doi:10.1093/ehjdh/ztag054)
Supplement: ztag054_Supplementary_Data [file ztag054_supplementary_data.zip › Supplement_Materials.docx]

**Supplementary Table 1. Hazard ratio at the end of the ablation procedure**

|  | | Univariate | | Multivariate | |
| --- | --- | --- | --- | --- | --- |
| Features | Hazard [95% CI] | | P | Hazard [95% CI] | P |
| Age per 10 | 0.99 [0.85–1.15] | | 0.93 | 0.97 [0.82–1.15] | 0.73 |
| Female | 0.71 [0.50–0.99] | | 0.05 | 0.72 [0.50–1.04] | 0.08 |
| BMI, kg/m^2^ | 1.01 [0.97–1.04] | | 0.77 | 0.97 [0.92–1.01] | 0.12 |
| LAD, mm | 1.05 [1.03–1.07] | | <0.01 | 1.05 [1.02–1.08] | <0.01 |
| Heart failure | 1.56 [1.15–2.13] | | <0.01 | 1.27 [0.91–1.79] | 0.16 |
| Hypertension | 0.85 [0.63–1.16] | | 0.31 | 0.83 [0.60–1.15] | 0.26 |
| Diabetes mellitus | 0.83 [0.56–1.23] | | 0.35 | 0.72 [0.48–1.08] | 0.11 |
| First session | 0.58 [0.41–0.83] | | <0.01 | 0.53 [0.37–0.76] | <0.01 |
| PAF | 0.63 [0.45–0.87] | | <0.01 | 0.77 [0.54–1.11] | 0.16 |
| Incomplete status assessed by AI | 2.17 [1.48–3.19] | | <0.01 | 1.95 [1.31–2.89] | <0.01 |

BMI: body mass index, LAD: left atrium diameter, PAF: paroxysmal atrial fibrillation, CI: confidence interval, and AI: artificial intelligence.

**Supplementary Table 2. Cox analysis in cases the AI assessed as ‘incomplete’**

|  | Univariate | | Multivariate | |
| --- | --- | --- | --- | --- |
| Features | Hazard [95% CI] | P | Hazard [95% CI] | P |
| Age per 10 | 0.92 [0.78–1.10] | 0.35 | 0.96 [0.78–1.18] | 0.70 |
| Female | 0.78 [0.50–1.22] | 0.28 | 0.81[0.50–1.32] | 0.41 |
| BMI, kg/m^2^ | 1.03 [0.99–1.08] | 0.20 | 1.00 [0.95–1.06] | 0.88 |
| LAD, mm | 1.03 [1.01–1.06] | 0.02 | 1.02 [0.98–1.05] | 0.35 |
| Heart failure | 1.66 [1.11–2.50] | 0.01 | 1.53 [0.98–2.38] | 0.06 |
| Hypertension | 0.87 [0.58–1.29] | 0.49 | 0.79 [0.51–1.22] | 0.29 |
| Diabetes mellitus | 1.05 [0.63–1.75] | 0.85 | 0.95 [0.56–1.60] | 0.85 |
| First session | 0.67 [0.43–1.03] | 0.07 | 0.60 [0.39–0.93] | 0.02 |
| PAF | 0.51 [0.34–0.78] | <0.01 | 0.62 [0.40–0.97] | 0.04 |
| Additional ablation | 0.56 [0.33–0.95] | 0.03 | 0.58 [0.34–0.99] | 0.05 |

BMI: body mass index, LAD: left atrium diameter, PAF: paroxysmal atrial fibrillation, and CI: confidence interval.

**Supplementary Table 3. Cox analysis in cases the AI assessed as ‘complete’**

|  | univariate | | multivariate | |
| --- | --- | --- | --- | --- |
| Features | Hazard [95%CI] | P | Hazard [95%CI] | P |
| Age per 10 | 0.99[0.87–1.14] | 0.91 | 0.99[0.85–1.15] | 0.86 |
| Female | 0.80[0.60–1.06] | 0.12 | 0.82[0.60–1.11] | 0.20 |
| BMI, kg/m^2^ | 0.99[0.96–1.03] | 0.68 | 0.96[0.92–1.00] | 0.05 |
| LAD, mm | 1.04[1.02–1.05] | <0.01 | 1.03[1.01–1.05] | <0.01 |
| Heart failure | 1.37[1.03–1.83] | 0.03 | 1.18[0.87–1.61] | 0.29 |
| Hypertension | 0.82[0.63–1.08] | 0.16 | 0.86[0.65–1.15] | 0.32 |
| Diabetes mellitus | 0.86[0.61–1.23] | 0.42 | 0.83[0.57–1.20] | 0.32 |
| First session | 0.54[0.42–0.71] | <0.01 | 0.50[0.36–0.70] | <0.01 |
| PAF | 0.69[0.52–0.91] | <0.01 | 0.76[0.56–1.03] | 0.08 |
| Additional ablation | 1.41[1.07–1.86] | 0.02 | 0.95[0.67–1.35] | 0.77 |

BMI: body mass index, LAD: left atrium diameter, PAF: paroxysmal atrial fibrillation, and CI: confidence interval.

**Supplementary Figure 1: Discriminative performance of ‘practice-based decision’**


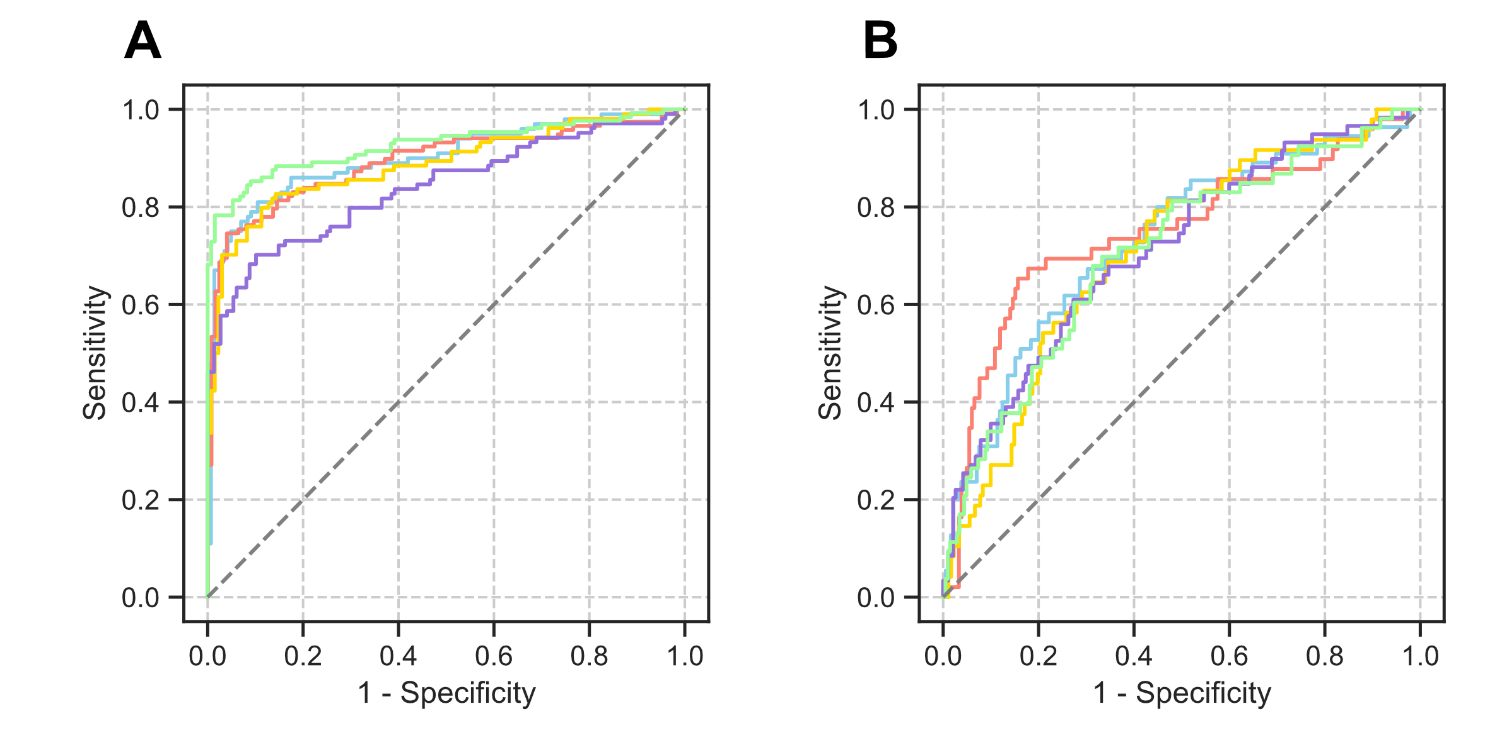


Evaluation of the discriminative performance of the ‘practice-based decision’ with fivefold cross-validation. **A** shows the time-dependent receiver operating characteristic curves of pulmonary vein isolation (PVI), and **B** shows those of beyond PVI (be-PVI).

**Supplementary Figure 2: Saliency maps of AI-based predictions for PVI and Be-PVI assessment**


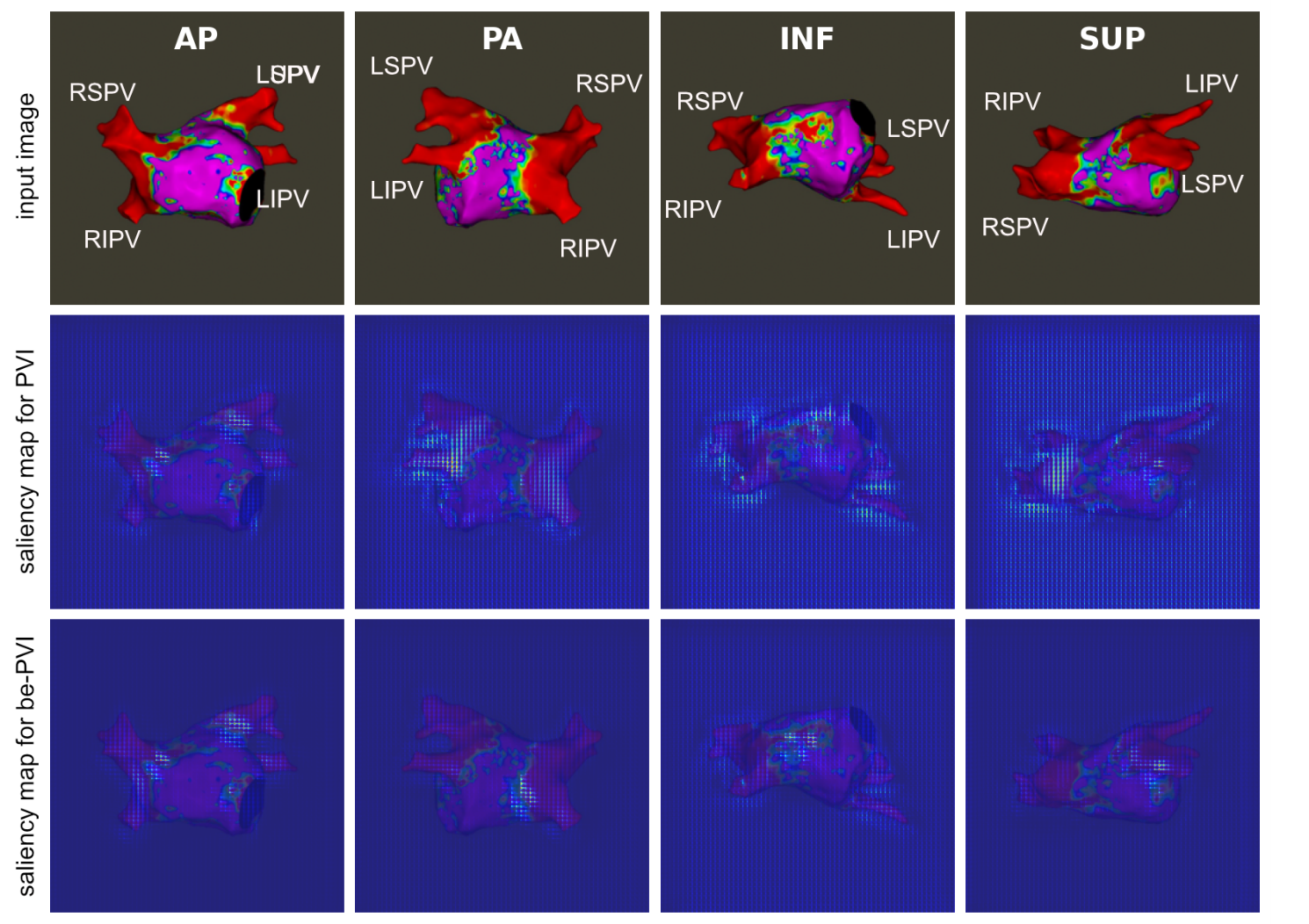


Visualisation of the AI model prediction with saliency maps produced via the SmoothGrad approach. From left to right: voltage maps from the anterior–posterior (AP), posterior–anterior (PA), inferior (INF), and superior (SUP) directions. The bright areas on the saliency map indicate the strong influence of the model on the prediction. Conversely, the dark blue areas minimally affected the prediction. The top panels are the original input images, the middle panels are the saliency maps that contribute to the prediction of pulmonary vein isolation (PVI), and the bottom panels are the saliency maps that contribute to the prediction of beyond PVI (be-PVI). RSPV: right superior pulmonary vein, RIPV: right inferior pulmonary vein, LSPV: left superior pulmonary vein, and LIPV: left inferior pulmonary vein.

**Supplementary Figure 3: Kaplan–Meier curves for AF recurrence risk stratified by the ‘practice-based decision’**


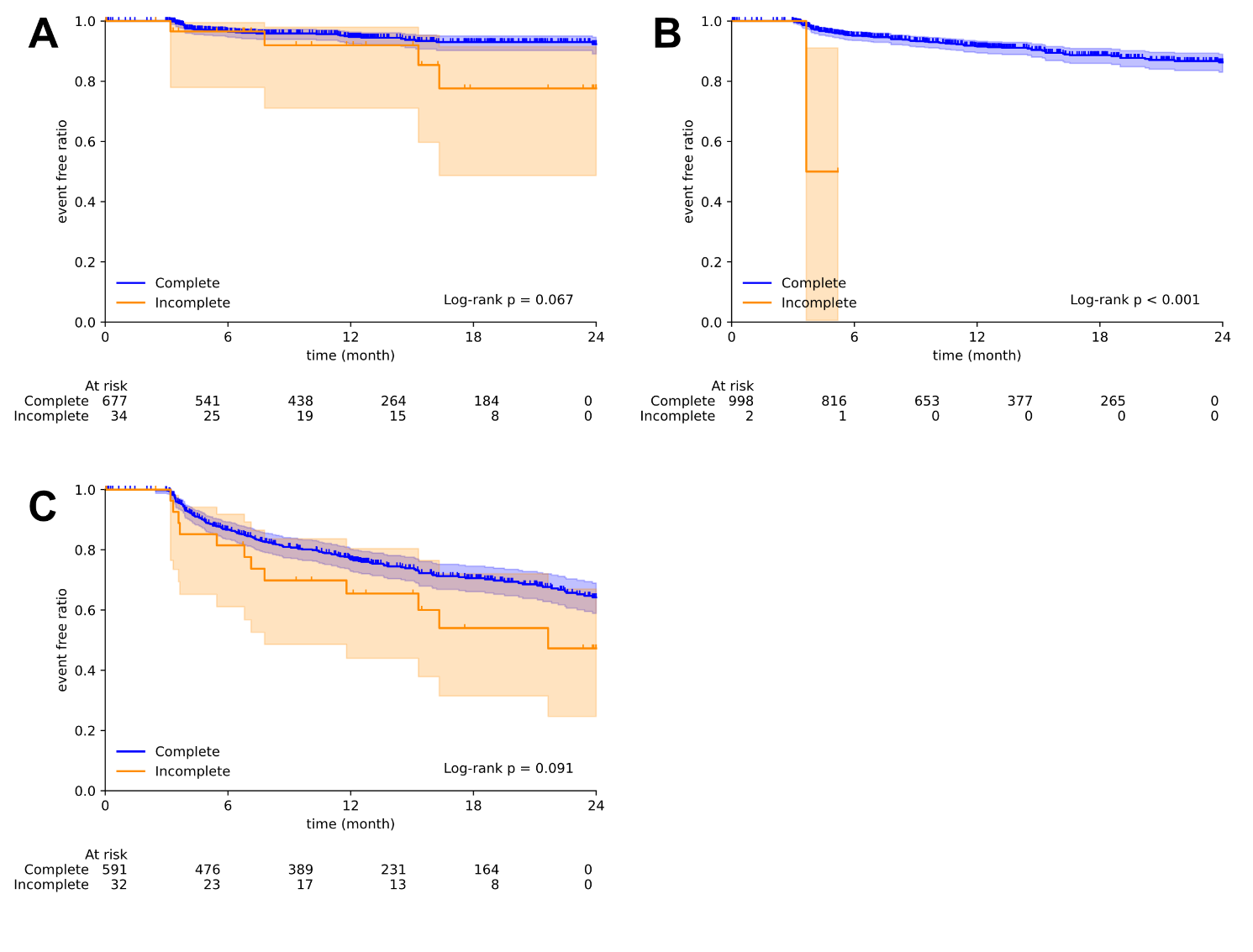


The AI performance for the ‘practice-based decision’ is evaluated using post-ablation voltage maps. Each panel shows performance for different recurrence definitions: AF recurrence requiring redo-PVI (**A**), redo-beyond-PVI (**B**), or any AF recurrence regardless of redo strategy (**C**). ‘Complete’ and ‘Incomplete’ indicate AI predictions of ablation status based on electrophysiological findings, independent of subsequent clinical outcomes.
